# Evolving Practices in Prenatal Open Spinal Dysraphism: A Global Survey of Selection Criteria, Surgical Techniques, and Diagnostic Trends

**DOI:** 10.1002/pd.70031

**Published:** 2025-11-26

**Authors:** Corinna Keil, Noemi Wiora, Eyal Krispin, Anita Windhorst, Roland Axt‐Fliedner, Ivonne Bedei

**Affiliations:** ^1^ Department of Prenatal Medicine and Fetal Therapy Philipps University Marburg Germany; ^2^ Department of Prenatal Diagnosis and Fetal Therapy Justus‐Liebig University Gießen Germany; ^3^ Boston Children's Hospital Brigham and Women's Hospital Harvard Medical School Boston Massachusetts USA; ^4^ Institute of Medical Informatics Justus‐Liebig University Gießen Germany

**Keywords:** fetal surgery, MOMS criteria, MOMS trial, open spinal dysraphism, spina bifida

## Abstract

**Objective:**

To provide an updated overview of international clinical practice in prenatal repair of open spinal dysraphism (OSD), focusing on evolving eligibility criteria, surgical techniques, and diagnostic standards.

**Methods:**

A structured online survey was distributed to 83 fetal surgery centers worldwide. The questionnaire addressed surgical techniques, maternal and fetal eligibility and diagnostic standards. Descriptive analyses were performed to identify current trends and practice variations.

**Results:**

38 centers from 16 countries participated in the survey (response rate 45.8%). Open fetal surgery remains the most common approach (51.4%) though 47.4% reported offering multiple techniques, including fetoscopic methods. Compared with the MOMS criteria, 42.4% performed surgery beyond 25.6 weeks of gestation, 52.4% accepted a BMI 35%–40% and 28.6% acc epted even a BMI of 41%–45%, and 42.4% treated women with prior uterine surgery. Most centers (87.9%) combined ultrasound and MRI for preoperative imaging. Genetic evaluation was heterogeneous: 66.7% required karyotyping, 63.6% required chromosomal microarray, 18.2% non‐invasive testing, and 6.1% required none. Prognostic indicators such as ventriculomegaly and motor function increasingly influence selection decisions.

**Conclusion:**

International practice in prenatal OSD repair shows broadening maternal eligibility, diversification of surgical approaches, and variable diagnostic strategies. These findings highlight a shift toward individualised care and emphasise the need for further studies to evaluate the impact of practice adaptations.

## Summary

- What is already known about this topic?
  ◦ Prenatal repair of open spinal dysraphism can improve neurological outcomes compared with postnatal repair. The MOMS trial established strict eligibility criteria, but real‐world practice has since diversified, with reports of deviations in maternal, fetal, and procedural selection.
- What does this study add?
  ◦ This international survey of 38 centers across 16 countries documents global practice patterns, highlighting procedural diversification, broader maternal eligibility, and evolving genetic testing approaches beyond the original MOMS framework.

## 1 Introduction

The Management of Myelomeningocele Study (MOMS), published in 2011, reshaped prenatal surgical strategies and influenced global practice, marking a pivotal moment in treating fetal spinal dysraphism. This randomized controlled trial demonstrated that prenatal repair significantly reduces the postnatal cerebrospinal fluid shunt rates and improves motor function in affected infants [1]. While establishing efficacy, it also underscored substantial maternal and fetal risks from intervention‐specific complications. Maternal morbidity, preterm birth, and obligatory Caesarean delivery for both the index and subsequent pregnancies remain major concerns. [1, 2, 3] The MOMS trial applied stringent inclusion and exclusion criteria, restricting eligibility according to specific feto‐maternal factors (Table 1). [1] Since MOMS, surgical innovations have sought to reduce maternal risks while maintaining or improving fetal outcomes [4, 5]. Minimally invasive techniques (minihysterotomy, laparotomy‐assisted fetoscopic ‐hybrid procedure, percutaneous fetoscopic or minilaparotomy assisted repair) have gained traction, by potentially reducing morbidity and preserving uterine integrity [6, 7]. However, these techniques are limited by prolonged operative times and challenges in achieving comparable closure quality [6, 8, 9].

**TABLE 1:** In‐ and exclusion criteria according to the randomized controlled Management of Myelomeningocele Study (MOMS) [1].

Inclusion criteria
Singleton pregnancy
Myelomeningocele with the upper boundary located between T1 and S1
Evidence of hindbrain herniation by MRI
A gestational age of 19.0–25.6 weeks at randomization
A normal karyotype
U.S. residency
Maternal age of at least 18 years
Major exclusion criteria
Multifetal pregnancy
Fetal anomaly unrelated to myelomeningocele
Severe kyphosis (of 30° or more)
Placenta praevia or placental abruption
Risk of preterm birth (including short cervix (< 20 mm) and previous preterm birth)
Current or planned cervical cerclage or documented history of cervical weakness
Body‐mass index of 35 or more
Insulin dependent pre‐gestational diabetes
Maternal hypertension that would increase the risk of pre‐eclampsia or preterm delivery
Maternal–fetal Rh isoimmunisation, kell sensitization or a history of neonatal alloimmune thrombocytopaenia
Maternal HIV or hepatitis‐B status positive, known hepatitis‐C positivity
Uterine anomaly such as large or multiple fibroids or mullerian duct abnormality
Contraindication to surgery, including previous hysterotomy in the active uterine segment
Patient does not have a support person
Inability to comply with the travel and follow‐up requirements of the trial
Patient does not meet other psychosocial criteria

Globally, many centers have adapted or extended the MOMS protocol to match patient populations, technical capabilities, or institutional preferences. This has included adjustments to gestational age thresholds, maternal weight, or the adoption of individualized surgical techniques tailored to the maternal‐fetal dyad [10, 11, 12, 13, 14]. Although no universally ‘ideal’ method exists, the variety of techniques highlights the need for individualised strategies based on maternal, fetal, and institutional factors [15].

The extent of deviation from the benchmark MOMS criteria and the rationale for such changes remain poorly characterised. To document the evolving landscape of prenatal OSD repair, we conducted a global survey of fetal surgery centers performing this intervention. The survey systematically captured deviations from the original protocol and the range of surgical techniques employed. By analyzing reported adaptations, this study provides a structured overview of international practice. While the underlying reasons for these modifications were beyond the scope of the survey, the findings offer insights into the diversity of approaches currently adopted.

## 2 Methods

### 2.1 Study Design

Between September 5^th^ and October 15^th^ 2024, we conducted an international cross‐sectional survey to assess contemporary clinical practices in centers offering prenatal repair of open spinal dysraphism (OSD), with a particular focus on deviations from the originally defined maternal and fetal eligibility criteria established in the Management of Myelomeningocele Study (MOMS) trial. The survey was developed in accordance with the Checklist for Reporting Results of Internet E‐Surveys (CHERRIES) guidelines [16]. Data were collected using the LimeSurvey platform (version 6.5.17).

The questionnaire in the English language consisted of structured items designed to evaluate deviations from the maternal and fetal benchmark inclusion criteria as described in the MOMS trial, as well as broader aspects of clinical decision‐making and surgical practice [1]. Data were collected using 5‐point Likert scales, single‐ and multiple‐choice formats, and open‐ended free‐text fields. Certain questions were mandatory for progression through the survey, while others permitted non‐response. For all predefined answer options, an “other” field was included to allow for additional specification. Participants were further invited to provide supplementary comments after the survey. The survey instrument underwent expert review and iterative refinement before dissemination (Supporting Information S1: Appendix 1‐ Survey on adherence to the MOMS criteria).

Eligible centers were defined as institutions that actively offered prenatal surgical treatment for OSD at the time of the survey. The initial identification of centers was based on the center listings reported by Sacco et al. and the subsequently updated ISPD “Fetal Therapy Centers Map” [17, 18]. This core list was supplemented by consultation of the North American Fetal Therapy Network (NAFTNet) Center Contact List and through independent online research conducted by the study team to ensure comprehensive global coverage [19].

This study involved the administration of an anonymous international survey among healthcare professionals, without the inclusion of patients or the collection of identifiable personal data. Following institutional policy and relevant regulatory definitions, the Ethics Committee of the Faculty of Medicine, Justus‐Liebig University Gießen, reviewed the study protocol the second of February 2024 and determined that it did not constitute research involving human subjects as defined under applicable ethical guidelines. Consequently, the study was classified as exempt from formal ethical approval and Institutional Review Board (IRB) review. The research was conducted in accordance with the principles outlined in the Declaration of Helsinki, and participants were informed about the voluntary and anonymous nature of their participation prior to survey initiation.

### 2.2 Statistical Analysis

For analysis purposes, responses were attributed to a single representative per center. In cases where multiple responses were received from the same institution, the most complete response was retained; duplicates were excluded. As the survey was conducted anonymously and without digital tracking, the voluntary identification of the responding center was used to minimize duplication bias. All data analyses were conducted using R statistical software (version 4.4.1) within the R Studio environment (version 2024.09.0). Descriptive statistics were used to summarize categorical variables as absolute frequencies and percentages. The primary outcome was the reported frequency and nature of deviations from the maternal and fetal MOMS inclusion criteria. To explore associations between center characteristics—such as the year of initiation of fetal spinal dysraphism surgery, annual case volume, and the number of different surgical techniques offered—and the extent of reported deviations from MOMS criteria, Fisher's Exact Test was applied due to the categorical nature of the data and the low expected frequencies in some subgroups. The significance threshold was set at *α* = 0.05.

## 3 Results

**FIGURE 1:**
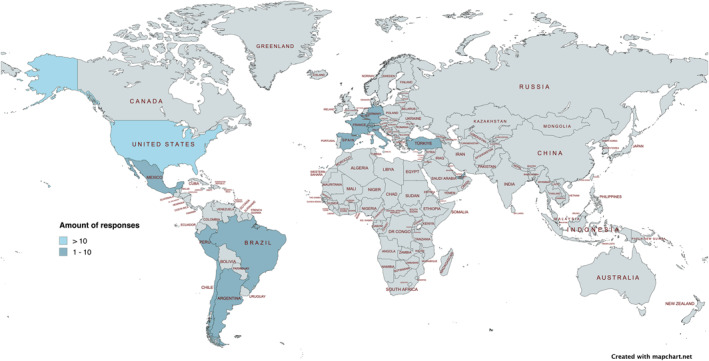
Geographic variation in survey responses. Created with mapchart.net. CC BY‐SA 4.0 (https://creativecommons.org/licenses/by‐sa/4.0/).

83 centers were identified as eligible and invited to participate. A total of 38 centers participated in the survey, yielding a response rate of 45.8%. Of these, 30 centers completed the full questionnaire. The geographic distribution of participating centers was as follows: United States (*n* = 12), Chile (*n* = 3), Spain (*n* = 2), and one center each from Argentina, Belgium, Brazil, France, Germany, Italy, Mexico, Peru, Switzerland, Turkey, and the United Arab Emirates (Figure 1). Data on institutional affiliation and country were missing for a subset of centers and are therefore not represented in the country‐level distribution. For all analyses, absolute numbers and percentages are reported relative to the number of centers that responded to the respective items. Consequently, denominators may vary across questions within the survey.

Centers were asked to report the year in which their prenatal OSD repair program was initiated; for the analysis, programs that commenced before 2017 were categorized as earlier initiations, with this threshold defined pragmatically based on the median initiation year (2017; range 2010–2024). Fifty percent of centers had established their programs by 2019, which also marked the peak year of program initiation (*n* = 7; 20.6%).

Survey responses were obtained from a range of medical specialties. Maternal–Fetal Medicine specialists constituted the largest group (57.9%), followed by specialists in Obstetrics and Gynecology (29%), Pediatric Surgery (13.2%), Pediatric Neurosurgery (10.5%), and Fetal Surgery (7.9%). Regarding operative involvement, maternal–fetal medicine specialists performed most reported surgeries (71.1%), followed by Neurosurgeons and Pediatric Neurosurgeons (55.2%) and Pediatric Surgeons (39.5%). Obstetricians were involved in 10.5% of the procedures.

### 3.1 Surgical Techniques and Institutional Experience

**FIGURE 2:**
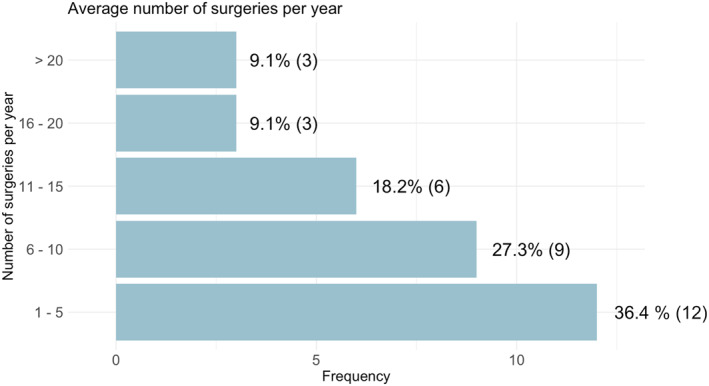
Average number of surgeries per year.

The number of fetal repair procedures performed per center within the preceding 2 years ranged from one to 200. Most centers reported conducting between one and five procedures per year (Figure 2). Open fetal surgery, as originally described in the MOMS study, represented the most performed technique, offered by 19/37 centers (51.4%). Laparotomy‐assisted fetoscopic repair and mini‐hysterotomy were performed in 14/37 centers (37.8%). Percutaneous fetoscopic surgery and mini‐laparotomy‐assisted fetoscopic repair were reported by 6/37 (16.2%) and 5/37 (13.5%) centers, respectively.

Among participating centers, 18/33 (54.5%) reported exclusive use of a single surgical approach, whereas 15/33 centers (45.5%) employed multiple techniques. Of these, 10 centers reported the use of two distinct approaches, and five centers implemented three or more techniques.

When centers were asked to specify the primary determinants influencing the choice of surgical technique for fetal spinal dysraphism repair, eight of 15 cited lesion type as the most decisive factor (53.3%). Additional factors, in descending order of frequency, included the patient's desire for future pregnancies (46.7%; 7/15), explicit maternal request (46.7%; 7/15), parity (46.7%; 7/15), maternal body mass index (26.7%; 4/15), placental location (2/15; 13.3%) and gestational age (GA) at surgery (1/15; 6.7%).

Regarding the GA window, most centers considered 23–24 weeks of gestation as the earliest GA threshold for fetal surgery, with 29 weeks as the upper limit. Notably, 42.4% (14/33) of centers indicated that they would sometimes, often, or always deviate from the GA inclusion criteria as defined in the MOMS trial.

### 3.2 Diagnostic Criteria for Fetal Surgery

Preoperative magnetic resonance imaging (MRI) to confirm fetal hindbrain herniation was deemed mandatory by 87.9% (29/33) of participating centers, who reported that they would rarely or never proceed without this diagnostic confirmation. In contrast, 12.1% (4/33) did not consider fetal MRI to be essential before surgery.

Concerning genetic testing before fetal surgery, conventional karyotyping was most frequently requested (66.7%; 22/33), followed by chromosomal microarray analysis (CMA) in 21/33 cases (63.6%) and Fluorescence in situ hybridisation (FISH) in 12/33 cases (36.4%). Two centers reported that they would not routinely request genetic testing (6.1%), whereas four would request exome sequencing (ES) (12.1%). When asked which genetic test would be required to be normal to proceed with fetal surgery, 19 of 33 centers (57.6%) required a normal karyotype, nine centers required a normal FISH result (27.3%) and 5 centers (15.1%) required CMA to be normal. No center required ES to be normal as mandatory before fetal surgery.

**FIGURE 3:**
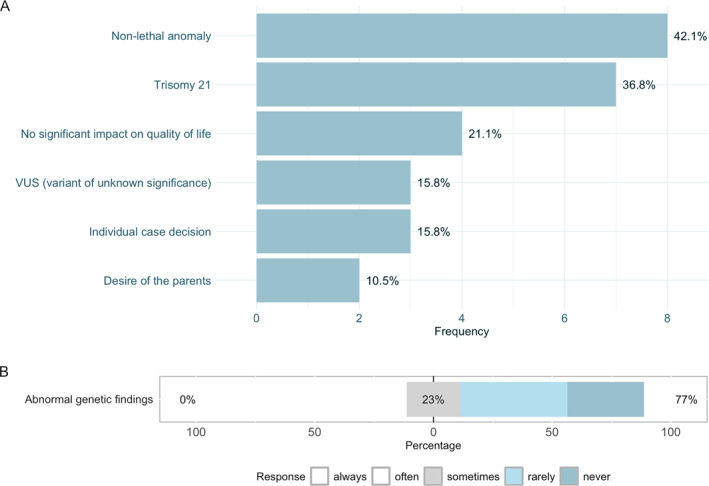
(A) Conditions and reasons to offer fetal surgery in case of an abnormal genetic finding. (B): Percentage of centers that would offer fetal surgery in case of an abnormal genetic finding.

A proportion of centers would nevertheless consider proceeding with surgery in cases of non‐lethal genetic conditions (e.g., trisomy 21), variants of uncertain significance (VUS), or based on explicit parental request when the genetic finding was not anticipated to have a substantial impact on postnatal quality of life (Figure 3A, B).

### 3.3 Lesion Characteristics and Fetal Anomalies as a Factor to Preclude Prenatal Surgery

**FIGURE 4:**
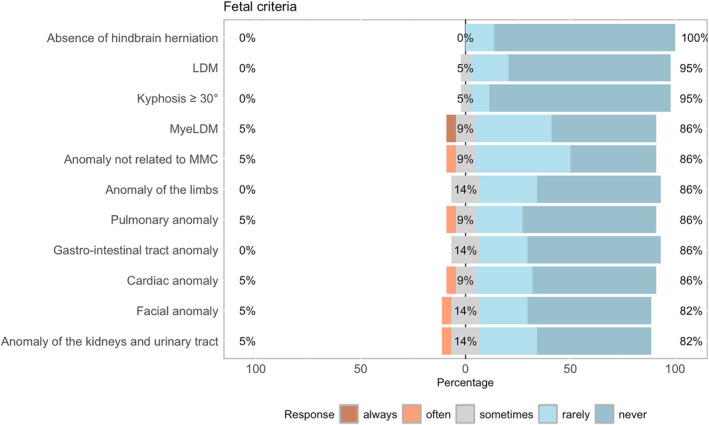
Deviation from fetal criteria. LDM, limited dorsal myeloschisis; MMC, myelomeningocele; MyeLDM, myelic limited dorsal malformation.

Hindbrain herniation was a mandatory criterion for centers offering fetal surgery. Large ventricular size, rupture of the cavum septum pellucidum, or severely impaired motor function would not preclude most centers from fetal repair. 27% would deviate from the anatomical level (t1‐S1). 5% would potentially operate on Limited Dorsal Myeloschisis (LDM), which is considered a closed form of spinal dysraphism, whereas 5% would always, and 9% would sometimes, operate on Myelic Limited Myeloschisis (MyeLDM). Fetal anomalies not related to spinal dysraphism would not be an exclusion criterion for 14% (Figure 4).

## 4 Maternal Criteria

**FIGURE 5:**
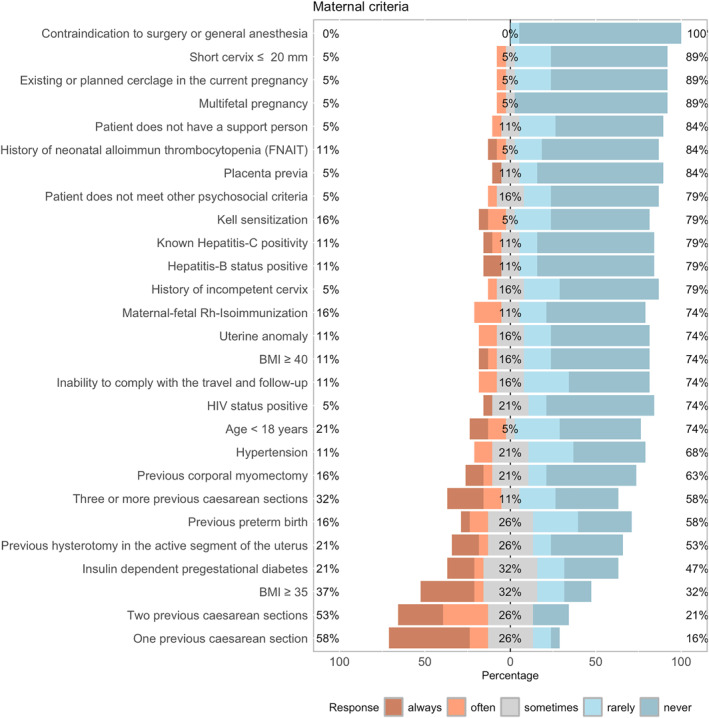
Deviation from maternal inclusion criteria.

Maternal characteristics and the proportions of deviations from the originally defined inclusion and exclusion criteria are presented in Figure 5. A statistically significant association was observed between earlier program initiation and deviations from the maternal eligibility criteria “previous corporal myomectomy” (*p* = 0.035) and “inability to comply with travel and follow‐up requirements” (*p* = 0.021). No significant association was found between program initiation year and deviations from the fetal MOMS criteria (*p* > 0.05).

No significant associations were identified between the number of surgical techniques offered per center and deviations from either maternal or fetal MOMS criteria (*p* > 0.05). In contrast, a significant relationship was found between a higher average annual surgical volume and deviations from the maternal criteria “BMI ≥ 35” (*p* = 0.005) and “Kell sensitization” (*p* = 0.049). No significant association was observed between surgical volume and deviations from the fetal MOMS criteria (*p* > 0.05).

Reasons for deviation from the MOMS inclusion and exclusion criteria were primarily based on individualized decision‐making and expert opinion, followed by evidence derived from cohort studies, case–control analyses, or interventional research.

## 5 Discussion

This international survey reports current practice in prenatal OSD repair, drawing on responses from 38 centers across 16 countries (45.8% response rate). Most centers were established after the MOMS trial, reflecting its global adoption. Open repair remains the most common technique though 47.4% of centers now offer multiple surgical approaches, including fetoscopic and hybrid procedures.

Although the MOMS criteria were widely cited, deviations were common. Specifically, 42.4% performed surgery beyond MOMS gestational limits, 63.6% accepted BMI ≥ 40, and 42.4% treated women with prior uterine surgery. In contrast, absolute contraindications—such as risks related to general anesthesia, multiple gestation, or active infection—were generally upheld. These findings indicate increasing individualization in maternal selection criteria, while core fetal criteria remain largely respected.

Our study shows wider global reach and greater diversification in OSD repair. While Sacco contacted 59 centers with a response rate of 74.6%, our survey reached 83 centers across a wider geographic distribution, although with a slightly lower response rate of 45.8% [17]. It is important to acknowledge that many fetal surgery programs, particularly in regions not traditionally covered in published registries or academic networks, may remain unidentified. These centers were not deliberately excluded but could not be captured by conventional data sources or earlier surveys. This highlights a structural limitation of current research approaches and supports the need for more comprehensive global registries [17, 20, 21, 22].

Building on the MOMS trial, new surgical techniques have emerged to achieve comparable benefits while reducing maternal and neonatal morbidity. Despite these procedural innovations, the original MOMS criteria continued to serve as a benchmarks [1, 23].

However, open fetal surgery remains the predominant technique, followed by laparotomy‐assisted fetoscopic repair and mini‐hysterotomy. Sacco's study primarily reflected open fetal surgery as the standard approach, whereas our data reveal a substantial increase in the use of fetoscopic techniques [17]. Furthermore, nearly half of the responding centers offer more than one surgical option, suggesting a shift toward individualized surgical planning.

The surgical approach is no longer strictly protocol‐driven; decisions are tailored to lesion anatomy, maternal preference, and reproductive planning. Prognostics considerations—such as the expected benefit based on motor function or ventricular size—now play a critical role in selecting both patients and surgical strategies.

Gestational age is another evolving criterion. While 14.7% of centers in Sacco's study exceeded the MOMS age limit, 42.4% did so in our study. Several factors may contribute to this shift. Logistical barriers such as delays in diagnosis, referral, or preoperative assessment may postpone surgery [11, 24, 25]. Additionally, some centers intentionally delay intervention to reduce the risk of extreme prematurity [13]. However, this approach remains debated. Some reports suggest that later gestational age does not necessarily worsen feto‐maternal outcomes, whereas others link it to a higher rate of ventriculoperitoneal shunt rates [4].

A further area of evolving practice concerns fetal imaging and genetic diagnostics. In the MOMS trial, both MRI confirmation of hindbrain herniation and a normal fetal karyotype were mandatory prerequisites. In our survey, most centers (87.9%) combined ultrasound and MRI, whereas 12.1% rely solely on ultrasound. Sonography remains the primary screening modality, but overestimates hindbrain herniation in approximately 5% of cases compared with MRI [26]. MRI also enhances surgical planning by enabling detailed evaluation of fetal (neuro‐) anatomy [27, 28].

In parallel, the landscape of prenatal genetic testing has expanded rapidly. Current professional guidelines recommend CMA as the first‐tier test in fetuses with major structural anomalies, including OSD [29, 30]. Whereas Sacco focused on karyotyping, our data show heterogeneity: most centers request karyotyping or CMA, some accept NIPT, and a few require no testing before surgery. Exome sequencing is requested by some centers but not a mandatory requirement in others.

This variability likely reflects national regulations, technology access, and reimbursement policies. Additionally, interpretation of abnormal findings—and their influence on eligibility for surgery—is modulated by ethical norms and legal frameworks, particularly regarding legislation on pregnancy termination. Nonetheless, comprehensive genetic assessment remains a crucial component of prenatal counseling, offering insight into prognosis, recurrence risk, and postnatal expectations [31, 32, 33].

Regarding feto‐maternal eligibility parameters, the survey confirms that these are no longer uniformly applied, with a distinct trend toward individualized assessment—particularly concerning maternal factors.

Fetal characteristics such as ventriculomegaly and motor function now influence decisions, though not specified in the MOMS protocol. Most centers do not exclude cases solely based on severe ventriculomegaly (> 15 mm), especially if motor function is preserved [34, 35, 36]. However, the combination of ventriculomegaly and motor impairment yields more variable responses. Additional anomalies unrelated to OSD were usually exclusionary, although some centers advocated case‐by‐case decisions [11, 37].

Maternal criteria showed the most variation. Elevated BMI, prior uterine surgery, or history of preterm birth are increasingly accepted, especially in high‐volume centers. We found that higher surgical volume correlated with acceptance of BMI ≥ 40 (*p* = 0.005), suggesting that experience shapes maternal eligibility thresholds [10, 38, 39]. Nonetheless, the inclusion of patients with additional maternal risk factors warrants attention from a patient safety perspective, particularly in low‐volume settings. Ongoing evaluation of selection criteria and structured training may help ensure consistent and safe application of prenatal repair.

These findings should be interpreted in light of several limitations. The response rate was modest (45.8%) and skewed toward Western institutions, which may limit global representativeness. As the data were self‐reported, they are subject to potential selection and reporting bias, and terms such as “frequent deviation” or “individualised approach” were not standardised, reducing comparability. The cross‐sectional design captures only one timepoint and cannot reflect evolving protocols, and no outcome data were linked to survey responses. Detailed information on specific intraoperative techniques, such as chorioamniotic membrane fixation, was not collected, as the study focused on variations in patient selection criteria rather than procedural aspects. Despite these considerations, a degree of selection bias due to the moderate response rate and regional overrepresentation cannot be excluded and should be considered when interpreting the results.

## 6 Conclusion

Our findings illustrate a pronounced trend toward flexible interpretation and contextual application of the accepted benchmark criteria. While the principles established by the MOMS trial remain a widely accepted reference, their implementation is increasingly modulated by institutional experience, procedural evolution, and local healthcare structures. Our data confirm the global spread of prenatal OSD repair and highlight a shift toward individualised, risk‐adapted decision‐making. This includes diversified surgical strategies, more nuanced approaches to genetic evaluation, and pragmatic adaptations to logistical and systemic constraints. These developments underscore the need to evaluate how deviations from the original criteria affect feto‐maternal outcomes in prenatal therapy [22, 40, 41].

## Funding

The authors have nothing to report.

## Ethics Statement

Following institutional policy and relevant regulatory definitions, the Ethics Committee of the Faculty of Medicine, Justus‐Liebig University Gießen, reviewed the study protocol the second of February 2024 and determined that it did not constitute research involving human subjects as defined under applicable ethical guidelines. Consequently, the study was classified as exempt from formal ethical approval and Institutional Review Board (IRB) review. The research was conducted in accordance with the principles outlined in the Declaration of Helsinki, and participants were informed about the voluntary and anonymous nature of their participation prior to survey initiation.

## Consent

This study involved the administration of an anonymous international survey among healthcare professionals, without the inclusion of patients or the collection of identifiable personal data.

## Conflicts of Interest

The authors declare no conflicts of interest.

## Supporting information


Supporting Information S1

## Data Availability

The data used to support this study's findings are available from the corresponding author upon reasonable request.
