# Supplementary material for: Evolving Practices in Prenatal Open Spinal Dysraphism: A Global Survey of Selection Criteria, Surgical Techniques, and Diagnostic Trends
Source: Prenat Diagn. 2025 Nov 26;46(1):75–83. doi: 10.1002/pd.70031 (PMC12819936; doi:10.1002/pd.70031)
Supplement: Supplementary file 1 — Supporting Information S1 [file PD-46-75-s001.docx]

# Survey on adherence to the MOMS criteria

1. Surgical procedures in the prenatal treatment of spina bifida
2. When did you start offering prenatal surgery for open spina bifida?
3. On average, how many surgeries do you perform at your center each year?

- 1-5
- 6-10
- 11-15
- 16-20
- > 20

1. Which surgical techniques are offered at your center?

(multiple answers possible)

- Open fetal surgery
- Open mini hysterotomy
- Percutanous laparoscopic fetal surgery
- Laparotomy assisted fetoscopic spina bifida repair (Hybrid procedure)
- Minilaparotomy assisted fetoscopic spina bifida repair
- Other:

1. Do you individually adapt the surgical technique to the patient?

|  | Never | Rarely | Sometimes | Often | Always | I don´t know |  |
| --- | --- | --- | --- | --- | --- | --- | --- |
|  |  |  |  |  |  |  | |

1. Which factors do you take into account when selecting the surgical procedure? (multiple answers possible)

- type of lesion (difficulty of repair/ extensive lesion)
- nulliparous
- multiparous
- maternal BMI
- maternal request
- desire for future pregnancy
- Other:

1. Do you consider different inclusion criteria to different surgical approaches?

|  | Never | Rarely | Sometimes | Often | Always | I don´t know |
| --- | --- | --- | --- | --- | --- | --- |
|  |  |  |  |  |  |  |

1. Requirements for prenatal surgery of spina bifida
2. How often do you deviate from the following criteria?

|  | Never | Rarely | Sometimes | Often | Always | I don´t know |  |
| --- | --- | --- | --- | --- | --- | --- | --- |
| Anatomical level T1 - S1 confirmed by ultrasound |  |  |  |  |  |  | |
| hindbrain herniation confirmed by MRI |  |  |  |  |  |  | |
| Gestational age at time of surgery between 19+0 and 25+6 weeks |  |  |  |  |  |  | |
| Normal FISH/karyotype |  |  |  |  |  |  | |

1. What is the minimum gestational age you perform fetal surgery?

[free text]

1. What is the maximum gestational age you perform fetal surgery?

[free text]

1. Do you offer fetal surgery based on prenatal ultrasound without confirmation of hindbrain herniation on MRI?

|  | Never | Rarely | Sometimes | Often | Always | I don´t know |
| --- | --- | --- | --- | --- | --- | --- |
|  |  |  |  |  |  |  |

1. Which genetic tests **do you request** before fetal surgery systematically? (multiple answers possible)
   - Noninvasive prenatal testing (NIPT) (trisomy 13/ 18/ 21)
   - Fluorescence in situ hybridization (FISH)
   - Karyotype
   - Chromosomal Microarray (CMA)
   - Exome sequencing (ES)
   - We do not request genetic testing before surgery
   - Other:
2. Which genetic test **is mandatory** to be normal before fetal surgery?
   - Noninvasive prenatal testing (NIPT) (trisomy 13/ 18/ 21)

- Fluorescence in situ hybridization (FISH)
- Karyotype
- Chromosomal Microarray (CMA)
- Exome sequencing (ES)
- We do not request genetic testing before surgery
- Other:

1. Would you consider offering open spina bifida repair in face of pathologic genetic findings?

|  | Never | Rarely | Sometimes | Often | Always | I don´t know |
| --- | --- | --- | --- | --- | --- | --- |
|  |  |  |  |  |  |  |

Please specify under which circumstances you would offer an open spina bifida repair in the event of a pathological genetic test:

1. Fetal criteria
2. How often do you decide to perform surgery when the following fetal criterion is present?

*assuming that all other MOMS criteria are met*

|  | Never | Rarely | Sometimes | Often | Always | I don´t know |
| --- | --- | --- | --- | --- | --- | --- |
| Anomaly not related to MMC |  |  |  |  |  |  |
| Anomaly of the kidneys and urinary tract |  |  |  |  |  |  |
| Facial anomaly |  |  |  |  |  |  |
| Cardiac anomaly |  |  |  |  |  |  |
| Digestive anomaly |  |  |  |  |  |  |
| Pulmonary anomaly |  |  |  |  |  |  |
| Anomaly of the limbs (not related to MMC) |  |  |  |  |  |  |
| Kyphosis ≥ 30° |  |  |  |  |  |  |
| Absence of hindbrain herniation |  |  |  |  |  |  |
| LDM |  |  |  |  |  |  |
| Hybrid cystic lesion (MyeLDM) |  |  |  |  |  |  |

1. What ventricular size would be considered an exclusion criterion?
   - 12mm
   - 15mm
   - 18mm
   - 20mm
   - None
2. Do you offer fetal surgery in the case of a rupture of the cavum septum pellucidum?

|  | Never | Rarely | Sometimes | Often | Always | I don´t know |
| --- | --- | --- | --- | --- | --- | --- |
|  |  |  |  |  |  |  |

1. What motor dysfunction would be considered an exclusion criteria?

- Club feet
- No knee movement
- No hips movement
- None

1. Maternal criteria
2. How often do you decide to perform surgery when the following maternal criterion is present?

*assuming that all other MOMS criteria are met*

|  | Never | Rarely | Sometimes | Often | Always | I don´t know |
| --- | --- | --- | --- | --- | --- | --- |
| Age < 18 years |  |  |  |  |  |  |
| Multifetal pregnancy |  |  |  |  |  |  |
| Insulin dependent pregestational diabetes |  |  |  |  |  |  |
| History of incompetent cervix |  |  |  |  |  |  |
| Existing or planned cerclage in the current pregnancy |  |  |  |  |  |  |
| Short cervix < 20 mm |  |  |  |  |  |  |
| Placenta previa |  |  |  |  |  |  |
| BMI ≥ 35 |  |  |  |  |  |  |
| BMI ≥ 40 |  |  |  |  |  |  |
| Previous spontaneous singleton delivery prior to 37 weeks of gestation |  |  |  |  |  |  |
| HIV status positive |  |  |  |  |  |  |
| Hepatitis-B status positive |  |  |  |  |  |  |
| Known Hepatitis-C positivity |  |  |  |  |  |  |
| Uterine anomaly such as large or multiple fibroids or mullerian duct abnormality |  |  |  |  |  |  |
| Previous hysterotomy in the active segment of the uterus |  |  |  |  |  |  |
| 1 previous caesarean section |  |  |  |  |  |  |
| 2 previous caesarean sections |  |  |  |  |  |  |
| 3 or more previous caesarean sections |  |  |  |  |  |  |
| Previous corporal myomectomy |  |  |  |  |  |  |
| Hypertension which would increase the risk of preeclampsia or preterm delivery |  |  |  |  |  |  |
| Maternal-fetal Rh-Isoimmunization |  |  |  |  |  |  |
| History of neonatal alloimmun thrombocytopenia (FNAIT) |  |  |  |  |  |  |
| Kell sensitization |  |  |  |  |  |  |
| Medical condition which is a contraindication to surgery or general anesthesia |  |  |  |  |  |  |
| Patient does not have a support person (e.g., husband, partner, mother) |  |  |  |  |  |  |
| Inability to comply with the travel and follow-up requirements of the trial |  |  |  |  |  |  |
| Patient does not meet other psychosocial criteria (as determined by the psychosocial interviewer using a standardized assessment) to handle the implications of the trial |  |  |  |  |  |  |
|  | Never | Rarely | Sometimes | Often | Always | I don´t know |

1. What is the minimum cervical length you perform fetal surgery?
2. What is the maximum BMI you perform open fetal surgery?
3. What is the maximum BMI you perform open mini hysterotomy?
4. What is the maximum BMI you perform percutanous laparoscopic fetal surgery?
5. What is the maximum BMI you perform laparotomy assisted fetoscopic spina bifida repair (Hybrid procedure)?
6. What is the maximum BMI you perform minilaparotomy assisted fetoscopic spina bifida repair?
7. Decision-making criteria
8. Which are the primary factors leading to your deviations from the MOMS criteria in your clinical practice? (multiple answers possible)
   - RCT
   - Expert opinion
   - Meta-analyses from several RCTs
   - Case-control studies
   - Controlled intervention study without randomization
   - Cohort study
   - Personal opinion
   - Individual (patient adapted) decision making
   - Other:
9. General information
10. Which surgical center do you work at?

center:

city:

country:

1. Which is your specialty? (multiple answers possible)

- Obstetrics and gynecology
- Maternal fetal medicine specialist
- Pediatric neurosurgeon
- Pediatric surgeon
- Neonatologist
- Other:

1. Who does fetal surgery at your center (multiple answers possible)

- Maternalfetal medicine specialist
- Obstetrician
- Pediatric neurosurgeon
- Pediatric surgeon
- Gynecologist/ Oncosurgeon
- Other

1. What ist the number of open spina bifida repairs done in your center at the past 2 years?
2. Additional notes
